# Phase II study of lanreotide autogel in Japanese patients with unresectable or metastatic well-differentiated neuroendocrine tumors

**DOI:** 10.1007/s10637-017-0466-8

**Published:** 2017-05-03

**Authors:** Tetsuhide Ito, Yoshitaka Honma, Susumu Hijioka, Atsushi Kudo, Akira Fukutomi, Akira Nozaki, Yasutoshi Kimura, Fuyuhiko Motoi, Hiroyuki Isayama, Izumi Komoto, Seiichi Hisamatsu, Akihiro Nakajima, Akira Shimatsu

**Affiliations:** 1 Department of Medicine and Bioregulatory Science, Graduate School of Medical Science, Kyushu University, Maidashi 3-1-1, Fukuoka Higashi-ku, Fukuoka, Japan; 2 Gastrointestinal Medical Oncology Division, National Cancer Center Hospital, Tokyo, Japan; 3 Department of Gastroenterology, Aichi Cancer Center Hospital, Nagoya, Japan; 4 Department of Hepato-Biliary-Pancreatic Surgery, Graduate School of Medicine, Tokyo Medical and Dental University, Tokyo, Japan; 5 Gastrointestinal Oncology, Shizuoka Cancer Center, Shizuoka, Japan; 6 Department of Medical Oncology, National Hospital Organization Kyoto Medical Center, Kyoto, Japan; 7 Department of Surgery, Surgical Oncology and Science, Sapporo Medical University Hospital, Sapporo, Japan; 8 Department of Surgery, Graduate School of Medicine, Tohoku University, Sendai, Japan; 9 Gastroenterology, The University of Tokyo Hospital, Tokyo, Japan; 10 Department of Surgery, Kansai Electric Power Hospital, Osaka, Japan; 11 Pharmaceutical Research & Development Division, Teijin Pharma Limited, Tokyo, Japan; 12 Clinical Research Institute, National Hospital Organization Kyoto Medical Center, 1-1 Mukaihata-cho, Fukakusa, Fushimi-ku, Kyoto, Japan

**Keywords:** Lanreotide, Neuroendocrine tumor, Japanese, Phase II, Somatostatin analog

## Abstract

*Background* Lanreotide is a long-acting somatostatin analog with demonstrated efficacy against enteropancreatic neuroendocrine tumor (NET) in the phase III (CLARINET) study. *Materials and Methods* In this single-arm study, Japanese patients with grade (G) 1/G2 NET received lanreotide (120 mg/4 weeks) for 48 weeks. Those who completed the study were enrolled in a long-term extension study. The primary endpoint was the clinical benefit rate (CBR) defined as a complete response, partial response (PR), or stable disease (SD) over 24-weeks. Secondary endpoints included progression-free survival (PFS), objective response rate (ORR), safety, and pharmacokinetics. *Results* Thirty-two patients were recruited at 10 sites. The full analysis set (FAS) comprised 28 patients. Primary tumors were located in pancreas (12 patients), foregut (non-pancreas, lung; 1), midgut (2), hindgut (8), and unknown (5). Four patients had gastrinoma of the functional NET, and 3 had multiple endocrine neoplasia type 1. In the FAS, 39.3% had progressive disease at baseline. The CBR at 24 weeks was 64.3% (95% confidence interval; CI: 44.1–81.4), and median PFS was 36.3 weeks (95% CI: 24.1–53.1). PR was confirmed in 1 patient at 60 weeks during the extension study (ORR: 3.6%). Frequent adverse events related to lanreotide included injection site induration (28.1%), faeces pale (18.8%), flatulence (12.5%), and diabetes mellitus (12.5%). *Conclusions* The efficacy and safety of lanreotide in this study indicated its usefulness as a treatment option for Japanese NET patients. Trial registration: JapicCTI-132,375, JapicCTI-142,698.

**Electronic supplementary material:**

The online version of this article (doi:10.1007/s10637-017-0466-8) contains supplementary material, which is available to authorized users.

## Introduction

Tumors occurring in nerves or endocrine cells anywhere in the body can be classified as neuroendocrine neoplasm (NEN). NEN is classified into poorly-differentiated neuroendocrine carcinoma and well-differentiated neuroendocrine tumor (NET). NET is a rare disease and tends to proliferate slowly. According to an epidemiological survey conducted in Japan in 2010, the incidence of new pancreatic and gastrointestinal NET was 4.78 per 100,000 people (estimated number of patients treated: 11,467) [1]. The incidences of NET other than pancreatic and gastrointestinal NET remain unknown in Japan, whereas the Surveillance, Epidemiology and End Results (SEER) Database [2] reported incidences of 2.85 and 2.15 per 100,000 people for pancreatic and gastrointestinal NET and other NET (lung, thymus, liver NET and NET of other/unknown origin), respectively. Although the only radical treatment for NET is surgical resection [3], approximately half of the patients present with tumor metastasis at the time of diagnosis [2] and therefore require pharmacotherapy. In Japan, the following drugs have been approved for specific types of NET: the molecular-targeted drugs everolimus for NET with all primary origins, sunitinib for pancreatic NET, the alkylating drug streptozocin for pancreatic and gastrointestinal NET, and the somatostatin analog octreotide for gastrointestinal NET. Lanreotide is a long-acting somatostatin analog. The CLARINET study [4], which was conducted in Europe and the USA, included 204 somatostatin receptor (sstr)-positive patients (193 Caucasian/White patients, 4 Black/African-American patients, and 7 Asian patients) with non-functional enteropancreatic NET (Ki67 index <10%). Statistically, lanreotide significantly prolonged the median progression-free survival (PFS) when compared with placebo (not reached vs. 18.0 months, *P* < 0.001; hazard ratio for PFS, 0.47 [95% CI: 0.30–0.73]) [4]. These anti-tumor proliferative effects led to the approval of lanreotide for enteropancreatic NET in the USA and European Union (EU). Additionally, lanreotide has been approved for the treatment of symptoms associated with NET (i.e., carcinoid syndrome) in more than 60 countries worldwide, including countries within the EU. We conducted a multicenter, single-arm phase II study to evaluate the efficacy, safety and pharmacokinetics of lanreotide in a population of Japanese patients with NET.

## Methods

### Study design and interventions

A phase II, multicenter, single-arm study was conducted at 10 sites in Japan. Patients were treated via deep subcutaneous (buttocks) injection of lanreotide at a dosage of 120 mg once every 4 weeks for a 48-week period. Patients who completed the phase II study were enrolled in an additional multicenter single-arm extension study that used the same dosage and injection methods and involved 7 of the 10 initial sites.

### Patients

Japanese patients with G1 or 2 (mild/moderate malignancy) NET and an age of ≥20 years were selected for the phase II study according to the World Health Organization (WHO) 2010 classification except for those whose primary tumors were located in the lungs, bronchi, or thymus (according to the WHO 2004 classification). Other inclusion criteria were the following: metastatic disease and/or a locally advanced inoperable tumor, or refusal of surgery, and target lesions based on the Response Evaluation Criteria in Solid Tumors (RECIST) ver.1.1, and a score of 0–2 on the WHO performance status. The exclusion criteria are described in the Electronic Supplementary Material. These criteria allowed the enrolment of the same target population as that of the CLARINET study: patients with G1 or 2 NET with primary tumor sites in the pancreas, midgut, hindgut, or unknown; non-functional NET except gastrinomas adequately controlled by proton pump inhibitors for 4 months or longer; and absence of multiple endocrine neoplasia.

In the extension study, all patients who completed the phase II study were eligible for entry. Inclusion was based on the investigator’s decision regarding the clinical indication of continued lanreotide injection.

### Assessment

The primary endpoint was the clinical benefit rate (CBR) at 24 weeks after the first injection, which served to indicate and confirm the anti-tumor effects of lanreotide. The CBR was defined as the percentage of patients in the analysis set who achieved a complete response or partial response (PR) as the best overall response, or those who maintained stable disease (SD) over 24 weeks after the first injection by central review. Secondary efficacy endpoints were progression-free survival (PFS), overall survival (OS), objective response rate (ORR), and the percent change from baseline in the sum of the diameters of target lesion from baseline. The PFS was defined as the time from the first injection until progressive disease (PD) or death. Regarding tumor lesion imaging ≥4 weeks before the baseline, the investigators evaluated computed tomography (CT) or magnetic resonance imaging (MRI) data based on the RECIST (ver. 1.1). To evaluate anti-tumor effects after baseline, CT or MRI data were evaluated centrally by an independent third party based on the RECIST (ver. 1.1). During the study period, images of each patient were determined using the same method (CT or MRI), according to the standardized procedure at each site. CT or MRI was conducted every 12 weeks from the first injection. Regarding quality of life (QOL), patients completed the European Organization for Research and Treatment of Cancer (EORTC) QOL questionnaire (QLQ)-C30 (ver. 3.0) every 12 weeks during the 48 -week period after the first injection and every 24 weeks thereafter.

For the safety evaluation, adverse events (AEs) were recorded after the first injection until the point of data cutoff (December 2015) in the extension study. AE severity was assessed according to the National Cancer Institute Common Terminology Criteria for Adverse Events (NCI-CTCAE) ver. 4.0. Gallbladder echography and blood evaluations, including serum anti-lanreotide antibody tests were performed every 12 weeks during the 48 -week period after the first injection, and every 24 weeks thereafter.

Serum CgA, serum anti-lanreotide antibody, and serum lanreotide testing methods are described in the Electronic Supplementary Material.

### Statistical analysis

Full analysis set (FAS) and CLARINET-like FAS which was satisfied eligibility criteria of the CLARINET study were used for efficacy analysis. Safety analysis set which included patients who had received the study drug at least once was used for safety analysis. Per protocol set (PPS) was used for pharmacokinetic analysis.

In the primary analysis, the CBR point estimate at 24 weeks after the first injection and an exact F distribution-based 95% confidence interval (CI) were calculated. The CBR was selected as the primary endpoint, because maintaining SD was deemed beneficial and clinically meaningful for NET patients. In the secondary analysis, Kaplan–Meier curves of PFS and OS were prepared, and median values and 95% CIs were calculated. The ORRs at 24, 48, and 60 weeks after the first injection were also calculated. Regarding percent changes from baseline in the sum of the diameters of target lesion, descriptive statistics were calculated for each evaluation time point, and waterfall plots of the best response and last evaluation time points were prepared. Regarding the serum CgA concentration and QOL evaluation, descriptive statistics of changes from baseline were calculated for each time point. For all endpoints, the starting point of evaluation was set as the baseline for the phase II study.

The descriptive statistics of tumor growth rate (TGR) [5] were calculated in a post hoc analysis of the FAS. The TGR estimates the change of the tumor volume during 1 month. This value incorporates the intervals between imaging tests, thus allowing a quantitative and dynamic evaluation of the tumor response. Scatter plots were prepared, and a subgroup analysis was conducted. To calculate TGR, results evaluated by the investigator were used before baseline, and results determined via central evaluation were used after baseline. There were not enough events for multivariate analysis, therefore univariate analysis based on the Cox proportional hazard model was implemented to assess prognostic factors.

The explanatory variables included age, sex, PD at baseline, prior treatment for NET, primary tumor site, grade, hepatic tumor load, Ki67 index, and baseline TGR.

## Results

### Patients

This phase II study included 32 patients who were treated with the study drug at 10 sites. According to the central assessment, 4 patients were excluded from the FAS (*n* = 28) due to the absence of target lesions. Seventeen of 32 patients (53.1%) completed the 48-week treatment period, whereas 15 patients (46.9%) discontinued the study.

All 17 patients who completed the phase II study (48 weeks, 12 times of injection) were enrolled in the extension study. Four out of 17 patients (23.5%) had discontinued the extension study at the time of data cutoff. The results of an analysis of pooled data from the phase II study and the extension study are shown below.

Baseline demographic and disease characteristics were evaluated in the FAS population. Eleven patients (39.3%) had PD at baseline. Twenty-two patients (78.6%) received prior treatment for NET. The primary tumor site was the pancreas in 12 patients (42.9%), foregut (except for pancreas; lung) in 1 patient (3.6%), midgut in 2 patients (7.1%), hindgut in 8 patients (28.6%), others/unknown in 5 patients (17.9%). Tumors were classified as G1 in 9 patients (32.1%) and G2 in 19 patients (67.9%). Although 4 patients (14.3%) had gastrinoma (functional NET), no patients exhibited hormone-related symptoms associated with NET at baseline (Table 1). Three patients (10.7%) were diagnosed as multiple endocrine neoplasia Type 1 (MEN1). No significant differences in baseline characteristics were observed between the FAS and CLARINET-like FAS (*n* = 22).

**Table 1:** Baseline demographic and disease characteristics (full analysis set, n = 28)

Age (years) ^*1^ Mean (standard deviation) Patients (%)		61.7 (11.3)
	≤65 years	16 (57.1)
	>65 years	12 (42.9)
Sex Patients (%)	Men	19 (67.9)
	Women	9 (32.1)
WHO performance status score Patients (%)	0 (Normal activity)	19 (67.9)
	1 (Restricted activity)	8 (28.6)
	2 (In bed <50% of the time)	1 (3.6)
Time after diagnosis (years)		
Mean (minimum value, maximum value)		6.33 (0.1, 23.3)
Presence or absence of progressive disease at baseline (RECIST standards) ^*2^ Patients (%)	Present	11 (39.3)
	Absent	17 (60.7)
Presence or absence of prior treatment for NET (drug or treatment method) Patients (%)	Present	22 (78.6)
	Absent	6 (21.4)
Classification of primary tumor site (based on embryological classification in CLARINET study) Patients (%)	Pancreas	12 (42.9)
	Foregut (except for pancreas)	1 (3.6)
	Midgut	2 (7.1)
	Hindgut	8 (28.6)
	Others/unknown	5 (17.9)
Grade of tumor ^*3^ Patients (%)	Grade 1	9 (32.1)
	Grade 2	19 (67.9)
	Grade 3	0
Ki67 index (%) Patients (%)	≤2	8 (28.6)
	>2 and ≤5	8 (28.6)
	>5 and <10	9 (32.1)
	≥10 and ≤20	2 (7.1)
	>20	0
	Unknown	1 (3.6) ^*4^
Number of mitosis (/ 10 HPF) Patients (%)	<2	3 (10.7)
	≥2 and <11	5 (17.9)
	≥11 and ≤20	1 (3.6)
	>20	0
	Unknown	19 (67.9)
Functional NET Patients (%)	Insulinoma	0
	Gastrinoma	4 (14.3)
	Glucagonoma	0
	VIPoma	0
	Somatostatinoma	0
	Tumors with carcinoid syndrome	0
	Others	0
Sum of the diameters of target lesion (mm) Mean (minimum, maximum)	≥4 weeks before baseline (determined by institution)	54.45 (12.8, 142.0)
	Baseline (determined by institution)	66.41 (14.1, 159.8)
	Baseline (central determination, RECIST Ver 1.1)	69.07 (29.6, 148.6)
Serum CgA concentration (ng/mL) at baseline		
Median (minimum value, maximum value) Patients (%)	127.4 (47.0, 10,557.6)	
	≤100	11 (39.3)
	>100 and ≤200	5 (17.9)
	>200	12 (42.9)
Hepatic tumor load (%)	≥0 and ≤10	18 (64.3)
Patients (%)	>10 and ≤25	6 (21.4)
	>25 and ≤50	3 (10.7)
	>50	1 (3.6)

*1: Age on the date of informed consent

*2: “Present” if the sum of the diameters of target lesion at baseline determined by the investigator on the basis of RECIST ver. 1.1 has increased by 20% or more as compared with more than 4 weeks before baseline, and “absent” if less than 20%

*3: Lung and bronchial origins were based on WHO 2004 classification. Other origins were based on WHO 2010 classification

*4: Reported as “lower than 10%” by the investigator

NET, neuroendocrine tumor; HPF, high-power field

### Efficacy

Efficacy was evaluated using results which obtained by the data in both the phase II and the extension studies. The CLARINET-like FAS consisted of 22 patients.

The CBR at 24 weeks after the first injection was 64.3% (18/28 patients; [95% CI: 44.1%, 81.4%]). The CBR at 24 weeks after the first injection in the CLARINET-like FAS was 68.2% (15/22 patients; [95% CI: 45.1%, 86.1%]).

**Fig. 1:**
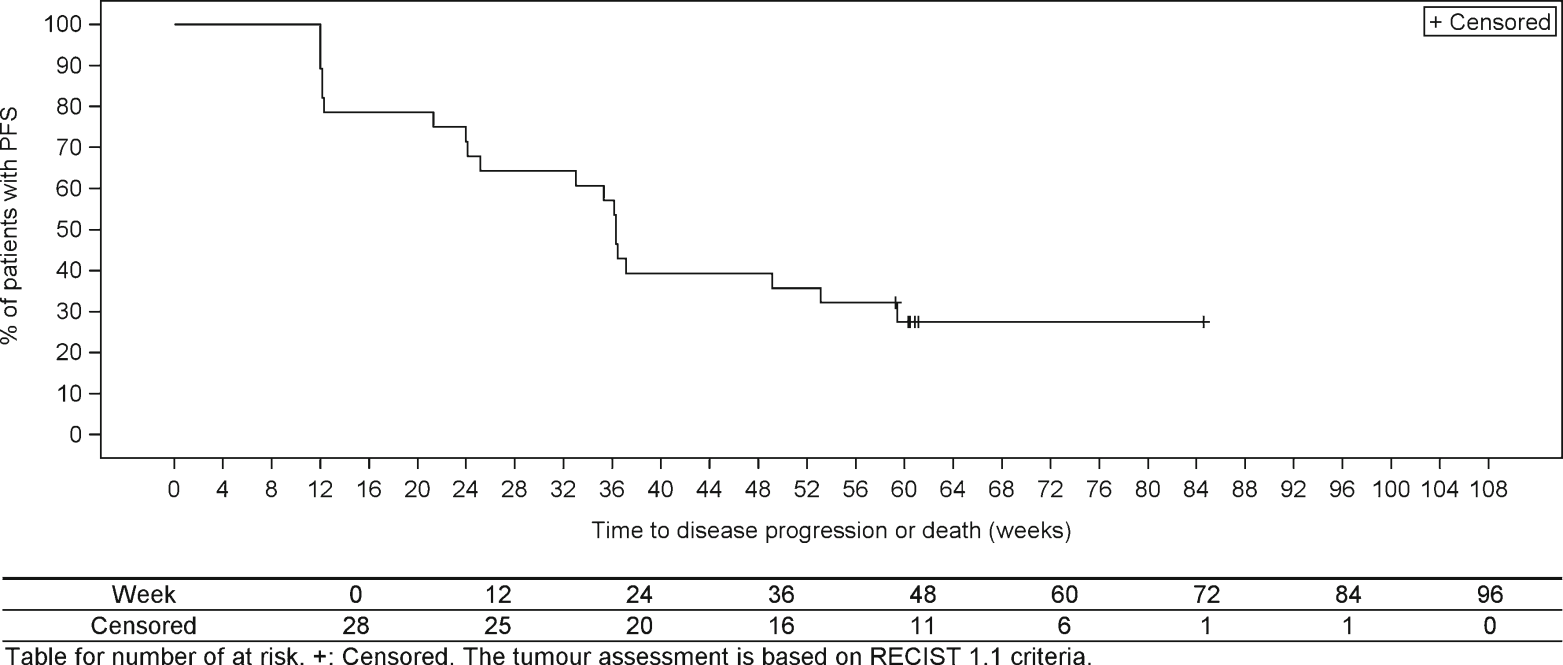
Kaplan-Meier analysis of progression-free survival (PFS; full analysis set, *n* = 28)

The median PFS in the FAS, estimated by the Kaplan–Meier method, was 36.3 weeks (9.1 months; [95% CI: 24.1 weeks, 53.1 weeks]; Fig. 1).

**Fig. 2:**
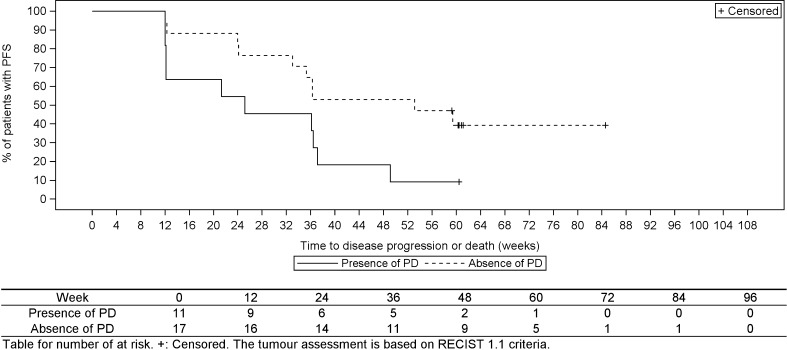
Kaplan-Meier analysis of progression-free survival (PFS) according to the presence or absence of progressive disease at baseline (full analysis set, *n* = 28)

The median PFS in the population with PD at baseline (11 patients) was 25.1 weeks (6.3 months [95% CI: 12.0 weeks, 37.1 weeks]). The median PFS in the population without PD at baseline (17 patients) was 53.1 weeks (13.3 months [95% CI: 24.1 weeks, Not calculable]) (Fig. 2).

Forty-eight weeks survival rate estimated by the Kaplan-Meier method was 96.4%, therefore the median OS was not reached in this study. Regarding the best overall response, 1 patient in the extension study achieved a PR at 60 weeks after the first injection, for an ORR at Week 60 of 3.6% (95% CI: 0.1%, 18.3%). Another patient failed to achieve PR but exhibited a percent decrease of ≥30% from baseline in the sum of the diameters of target lesion at 24 weeks after the first injection.

**Fig. 3:**
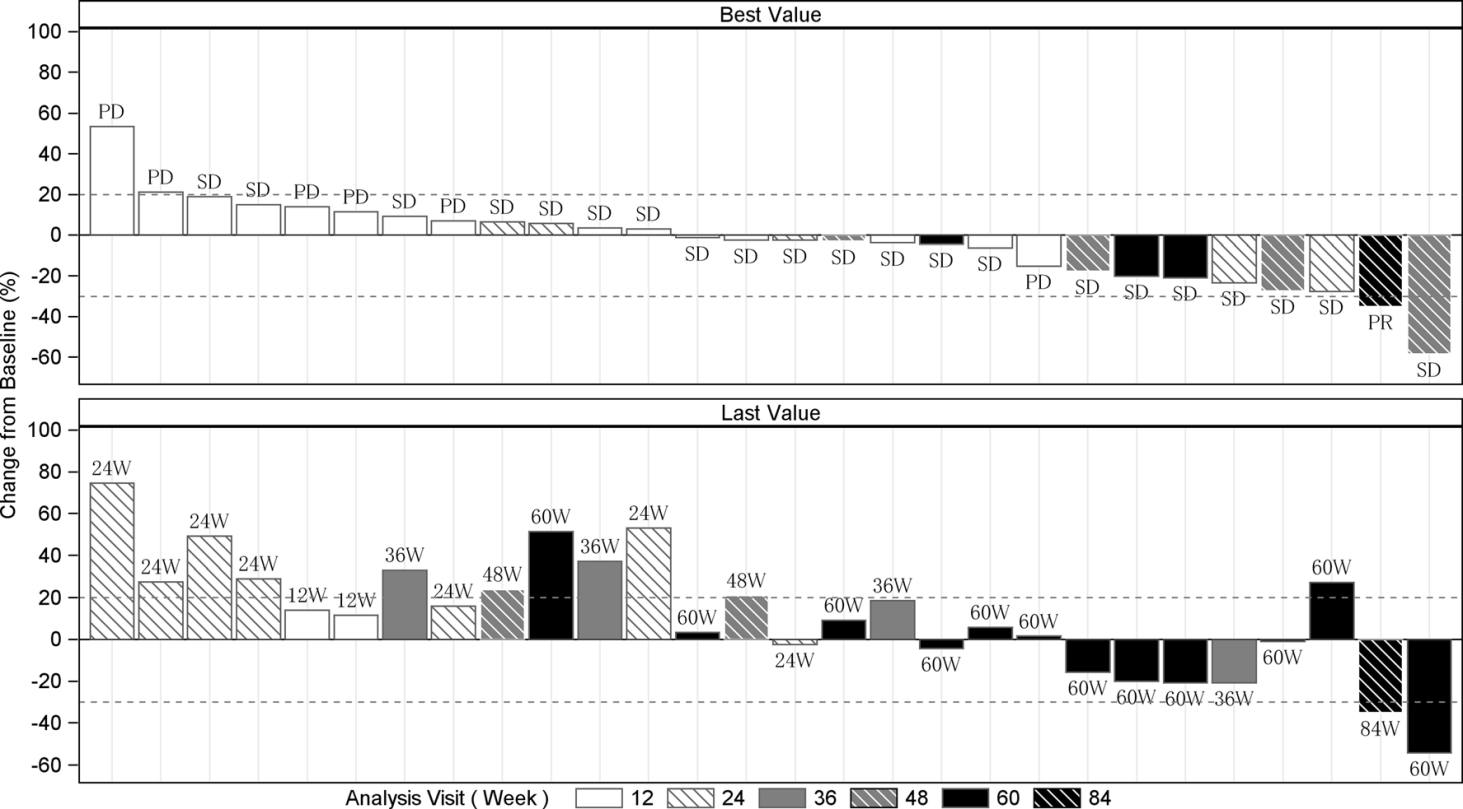
The best and the last percent change from baseline in the sum of the diameters of target lesion by patient (full analysis set, *n* = 28). Note: The best overall response results are shown in the bar graph at the top, and the numbers of weeks at the time point of the last observation are shown at the bottom. The study duration at the last observation is shown as the last value in the bar graph. Bars corresponding to results from the same patient are located in the same rows in the top and bottom charts

The mean (± SD) of the percentage change from baseline in the sums of the diameters of target lesion per patient at the time of the best percent change from baseline (best time point) decreased by 3.6 ± 21.3%, the minimum value decreased by 58.5%, and the maximum value increased by 53.4% (Fig. 3). Overall, 16 patients (57.1%) did not exhibit an increase in the sum of the diameters of target lesion at the best time point, and 22 patients (78.6%) achieved a PR or SD as the best overall response.

Among the 28 patients in the FAS, 17 (60.7%) had serum CgA concentrations >100 ng/mL at baseline. All 17 patients exhibited decreases in this parameter after the first injection. Among them, 15 patients (88.2%) exhibited decreases of ≥50% by the time of the last examination relative to the baseline.

In the QOL assessment, the transformed scores did not differ significantly from the baseline in individual terms.

### Post hoc analysis

**Fig. 4:**
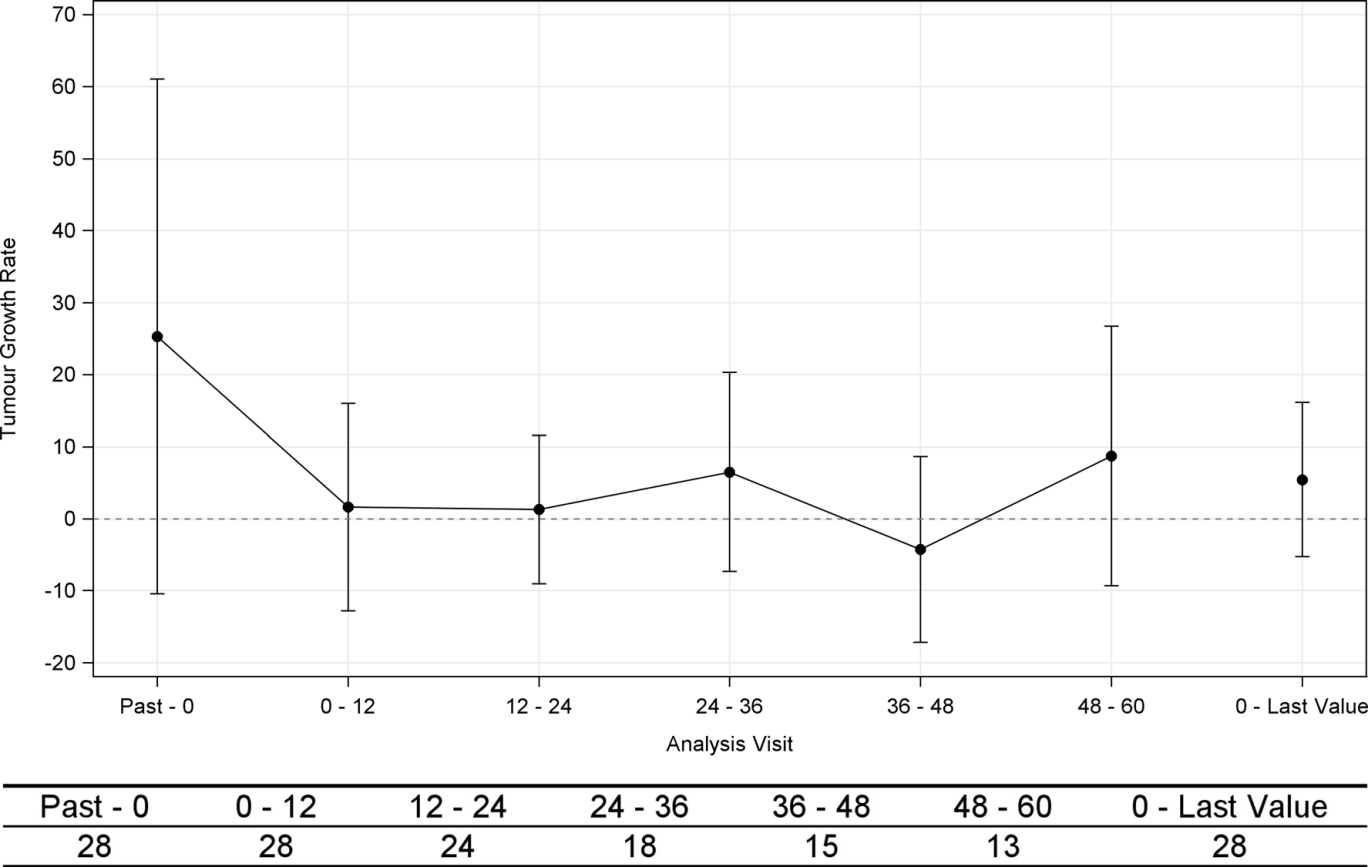
Percent changes from baseline in mean (± standard deviation) tumor growth rates (TGR; full analysis set, *n* = 28). Note: Data from Past to 0 (baseline) were evaluated by the investigator based on RECIST (ver. 1.1). Data after the baseline were evaluated based on RECIST (ver. 1.1) via central assessment by an independent third party

The TGR per unit time was calculated in the post-hoc analysis. From past to 0 (baseline), the results of the investigator’s evaluation were used for tumor lesions, after baseline, the results of a central evaluation were used for the tumor lesions. The TGR (mean ± SD) before the first injection was 25.3 ± 35.7%, and the TGR from baseline to the last value was 5.4 ± 10.7% (Fig. 4).

**Fig. 5:**
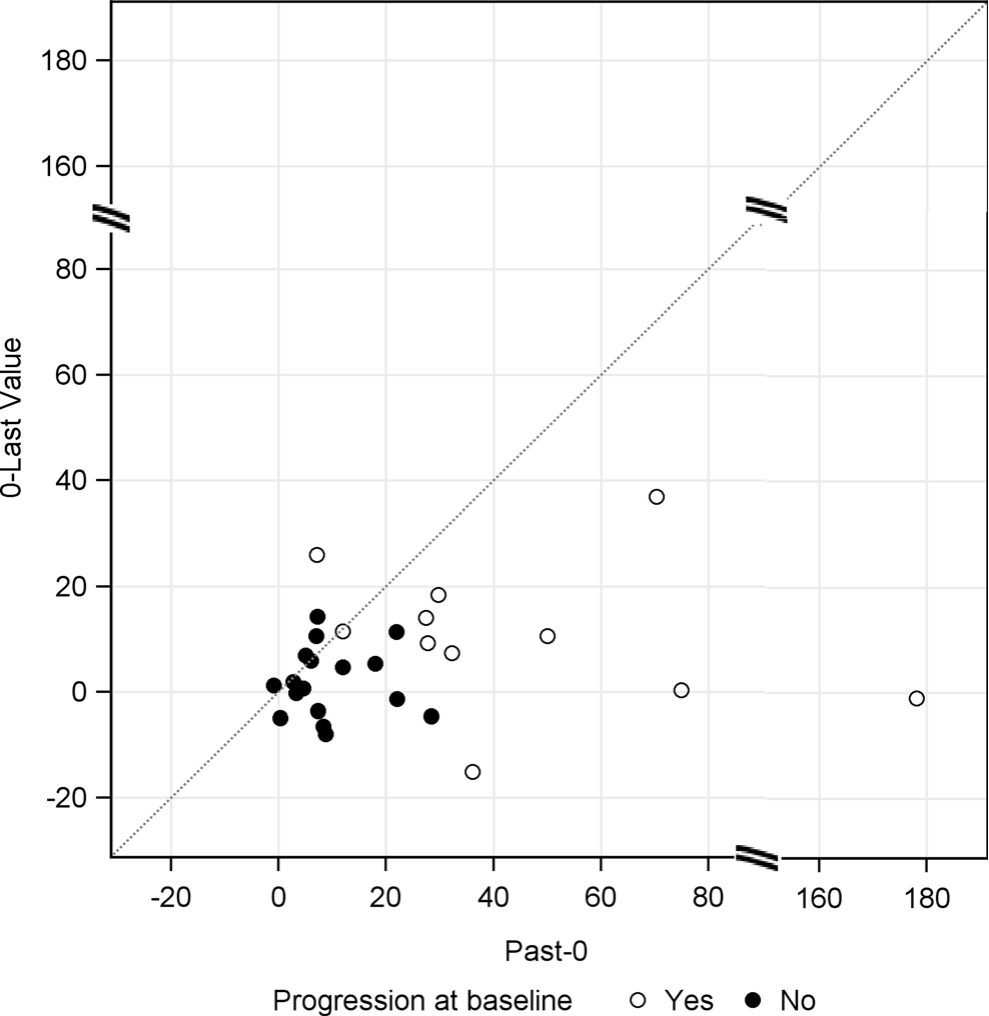
Scatter plot of tumor growth rates (full analysis set, *n* = 28)

In a scatter plot of TGRs stratified by the presence or absence of PD at baseline, the TGR mostly decreased at the last value relative to its value before the first injection, irrespective of the presence or absence of PD at baseline. Among the population with PD at baseline (11 patients), 10 (90.9%) exhibited decreases in TGR at the last value (Fig. 5).

In univariate analysis of PFS, PD at baseline (RECIST criteria, local), prior treatment for NET, Ki67 index, and baseline TGR were statistically significant with respect to PFS at a significance level of 10% (Table 2). These variables were thought to be prognostic factors of disease progression.

**Table 2:** Results of a univariate analysis of PFS

	Subgroup		Median time PFS (weeks)	Cox Hazard Model		CBR Week 24	TGR (0-Last)
Subgroup	Category	N	Median (95% CI)	HR (95% CI)	*P*-Value	% (n/N)	Mean ± SD
Age (years)	≤65	16	36.29 (12.29, 53.14)	1.00	0.632	68.8 (11/16)	5.41 ± 11.34
	>65	12	36.64 (12.14, NC)	0.80 (0.32, 1.98)		58.3 (7/12)	5.44 ± 10.34
Sex	Male	19	36.14 (12.29, 49.14)	1.00	0.428	63.2 (12/19)	6.71 ± 11.07
	Female	9	53.14 (12.00, NC)	0.68 (0.26, 1.78)		66.7 (6/9)	2.71 ± 10.00
Presence or absence of progressive disease at baseline (based on RECIST standards)	Present	11	25.14 (12.00, 37.14)	2.54 (1.04, 6.20)	0.041	45.5% (5/11)	10.74 ± 13.84
	Absent	17	53.14 (24.14, NC)	1.00		76.5% (13/17)	1.99 ± 6.50
Presence or absence of prior treatment for NET (drug or treatment method)	Present	22	36.22 (12.29, 49.14)	3.99 (0.91, 17.40)	0.066	54.5 (12/22)	6.72 ± 11.55
	Absent	6	NC (33.00, NC)	1.00		100.0 (6/6)	0.69 ± 5.09
Classification of primary tumor site (based on embryological classification in CLARINET study)	Pancreas	12	36.79 (24.14, 59.43)	1.00	0.615	83.3 (10/12)	0.43 ± 7.83
	Foregut (except for pancreas)	1	12.00 (NC, NC)	960,261.8 (0.00, NC)		0.0 (0/1)	0.32 ± NC
	Midgut	2	NC (NC, NC)	0.00 (0.00, NC)		100.0 (2/2)	-2.77 ± NC
	Hindgut	8	22.65 (12.14, 49.14)	2.19 (0.80, 6.00)		25.0 (2/8)	16.35 ± 11.02
	Other/ Unknown	5	36.14 (12.00, NC)	0.97 (0.26, 3.58)		80.0 (4/5)	4.23 ± 6.12
Grade of tumor	Grade 1	9	NC (12.00, NC)	1.00	0.195	55.6 (5/9)	1.65 ± 5.95
	Grade 2	19	36.29 (21.29, 49.14)	2.07 (0.69, 6.25)		68.4 (13/19)	7.21 ± 12.09
Ki67 index (%)	≤ 2	8	NC (12.00, NC)	1.00	0.016	62.5 (5/8)	1.82 ± 6.34
	>2 and ≤5	8	42.72 (12.00, NC)	1.85 (0.44, 7.75)		62.5 (5/8)	5.55 ± 8.50
	>5 and <10	9	36.29 (21.29, 53.14)	3.22 (0.85, 12.15)		88.9 (8/9)	4.63 ± 11.81
	≥10 and <20	2	12.07 (12.00, 12.14)	46.04 (4.37, 484.62)		0.0 (0/2)	7.19 ± NC
	Unknown	1	12.14 (NC, NC)	28.29 (1.95, 410.48)		0.0 (0/1)	36.90 ± NC
Hepatic tumor load (%)	≥0 and ≤10	18	36.36 (21.29, 59.43)	1.00	0.835	66.7 (12/18)	4.09 ± 9.60
	>10 and ≤25	6	35.79 (12.00, NC)	1.01 (0.33, 3.10)		66.7 (4/6)	8.10 ± 15.47
	>25 and ≤50	3	25.14 (24.14, 37.14)	1.81 (0.50, 6.55)		33.3 (1/3)	9.49 ± 9.98
	>50	1	NC (NC, NC)	0.00 (0.00, NC)		100.0 (1/1)	1.17 ± NC
Baseline TGR (%/month)	<10	13	59.43 (24.00, NC)	1.00	0.078	76.9 (10/13)	3.41 ± 9.43
	≥10	15	35.29 (12.14, 37.14)	2.31 (0.91, 5.83)		53.3 (8/15)	7.17 ± 11.76

This analysis of PFS time considers as events centrally assessed disease progressions (using RECIST 1.1 standards) and any deaths reported during the study. PFS, progression-free survival; CBR, clinical benefit rate; TGR, tumor growth rates; CI, confidence interval; HR, hazard rate; SD, standard deviation; NC, not calculated; RECIST, Response Evaluation Criteria in Solid Tumors

### Safety

Safety was evaluated using results which obtained by the data in the phase II and extension studies up to the time of data cutoff. The maximum exposure period from baseline to the time of data cutoff was 100.0 weeks (median, 87.3 weeks), and the mean exposure period (SD) was 83.1 weeks (15.2 weeks).

AEs were observed in 31 patients (96.9%). AEs related to study drug reactions (ADRs) were observed in 27 patients (84.4%). Frequent ADRs included injection site induration (28.1%), faeces pale (18.8%), flatulence (12.5%), and diabetes mellitus (12.5%) (Table 3). Grade 3 or higher AEs (NCI-CTCAE ver. 4.0) are listed in the eTable 1 (Electronic Supplementary Material). Grade 3 or higher ADRs accounted for 18.8% (Grade 3 only). The following events occurred in 6 patients: abdominal pain upper, pancreatitis, diabetes mellitus, hyperglycaemia, diabetes mellitus inadequate control, blood glucose increased, and hypertension).

**Table 3:** Adverse events related to the study drug occurring in ≥5% of patients (safety analysis set)

SOC ^1)^ PT	Safety analysis set (*n* = 32)
Any AE related to the study drug	**27 (84.4)**
Gastrointestinal disorders	15 (46.9)
Faeces pale	6 (18.8)
Flatulence	4 (12.5)
Abdominal distension	3 (9.4)
Abdominal pain	2 (6.3)
Constipation	2 (6.3)
Diarrhoea	2 (6.3)
Nausea	2 (6.3)
General disorders and administration site conditions	**13 (40.6)**
Injection site induration	9 (28.1)
Injection site pruritus	3 (9.4)
Malaise	3 (9.4)
Injection site pain	2 (6.3)
Pyrexia	2 (6.3)
Metabolism and nutrition disorders	**7 (21.9)**
Diabetes mellitus	4 (12.5)
Hyperglycaemia	2 (6.3)
Investigations	**4 (12.5)**
Alanine aminotransferase increased	3 (9.4)
Aspartate aminotransferase increased	3 (9.4)

SOC, system organ class; PT, preferred term

^1)^MedDRA version 16.0 was used for coding

Serious AEs occurred in 6 patients (18.8%). Among them, pyrexia and bile duct stone (3.1% each) were regarded as serious ADRs.

Anti-lanreotide antibody data are provided in the Electronic Supplementary Material.

### Pharmacokinetics

Pharmacokinetics outcomes were analyzed in the PPS (28 patients) based on results obtained in the phase II study. The serum lanreotide concentration after the first injection reached a maximum blood concentration (C_max_) of 20.2 ± 18.2 ng/mL (mean ± SD) at 3.9 h (median) after the first injection and gradually decreased to 3.1 ± 1.4 ng/mL before the first injection at 4 weeks after the first injection. The C_max_ at 20 weeks after the first injection was 24.2 ± 20.2 ng/mL, the trough concentration at 24 weeks after the first injection was 6.1 ± 3.3 ng/mL, and the median time to reach the maximum serum concentration was 3.8 h. Concentrations reached a near- steady state after 3 doses.

## Discussion

This is the first report of results from a clinical study that evaluated the efficacy (i.e., antitumor effects and PFS), safety, and pharmacokinetics after treatment with somatostatin analogs in the population of Japanese patients with G1/2 NET originating from the pancreas, gastrointestinal tract, or lungs. The antitumor effects of somatostatin analogs are known to comprise both direct (cellular growth inhibition via sstr [6] and apoptosis [7]) and indirect actions [8] (inhibition of growth factor secretion). This study evaluated the efficacy of the sustained-release lanreotide formulation in 28 patients in the FAS. While the data are limited by the small sample size, antitumor activity was observed in this population. The CBR at Week 24 was 64.3%, PR was established in 1 patient (ORR 3.6%), and the median PFS was 36.3 weeks (9.1 months).

Regarding safety, gastrointestinal disorders were the most commonly-observed ADRs associated with injection of the study drug per the protocol. This finding was consistent with the known safety profile of this drug. Grade 3 ADRs were observed in 6 patients (18.8%). Grade 4/5 ADRs and ADRs necessitating discontinuation of the study drug did not occur.

Regarding pharmacokinetics, the mean values (± SD) of trough concentrations during the treatment period ranged from 3.1 ± 1.4 to 6.1 ± 3.3 ng/mL, and appeared to reach a steady state after 3 injections. In the CLARINET study, the trough concentrations during the treatment period ranged from 2.5 ± 1.1 to 6.9 ± 3.0 ng/mL, and appeared to reach a steady state after 6 injections. No interracial differences were observed regarding drug concentrations in the blood. This inter-study difference in the number of treatment required to achieve steady state is attributed to the associated with the low number of blood collection points in the CLARINET study.

Furthermore, in the CLARINET study, a median PFS was not reached at 96 weeks (24 months) [4], in contrast to the median PFS of 36.3 weeks (based on the RECIST ver. 1.1) in the present study. This difference is attributed to differences in baseline disease characteristics of the patient and tumor characteristics. The percentages of patients with PD at baseline and prior treatment for NET were 39.3% (11/28 patients) and 78.6% (22/28 patients), respectively, in the present, and only 4.0% (4/101 patients) and 15.8% (16/101 patients), respectively, in the CLARINET study. The current Japanese study was initiated after the results of the CLARINET study were published and the inhibitory effects of lanreotide on tumor proliferation were revealed. Furthermore, these studies differed regarding the therapeutic management of the NET before study entry. Accordingly, differences in the patients’ backgrounds might have affected median PFS. However, no major difference in PFS was observed if the subjects’ baseline disease characteristics were adjusted in a stratified analysis (Fig. 2).

In the post-hoc analysis, the TGR per unit period was calculated for each patient. In contrast to RECIST, the TGR enables an evaluation of monthly tumor growth and it is thought to enable a more dynamic evaluation of treatment effects [5]. As shown in Figs. 4 and 5, patients in the FAS tended to exhibit a reduced TGR after lanreotide injection. Moreover, the 10 of 11 patients (90.9%) with PD at baseline exhibited decreases in TGR. The TGR remained stable among patients with SD at baseline.

The post-hoc univariate analysis of PFS revealed, statistically significant differences depending on the baseline status (progressive or stable), prior treatment for NET, Ki67 index, and baseline TGR. The PD at baseline was also suggested to be a prognostic factor as a result of the multivariate analysis in the CLARINET study [9].

In the CLARINET study, the sustained-release lanreotide formulation was administered to a wider variety of patients, compared to the octreotide sustained release formulation [10], (both G1 and G2, pancreas/hindgut and midgut, hepatic tumor load >10%), and efficacy and safety were evaluated. Similar results were also observed in the phase II study of Japanese patients. Moreover, the sustained-release lanreotide formulation has been associated with less severe ADRs (e.g., interstitial pneumonia or leukocytopenia), compared to molecular-targeted drugs. ADRs that occurred frequently in this study included injection site induration in 9/32 patients (28.1%) and faeces pale in 6/32 patients (18.8%). Our safety profile agreed with those of past clinical studies of lanreotide, and no difference was observed when the safety profiles were compared in patients with non-NET diseases (e.g., acromegaly). The sustained-release lanreotide formulation is expected to exhibit prolonged efficacy even when administered as a monotherapy, without necessitating drug discontinuation because of ADRs.

In conclusion, the efficacy and safety results of lanreotide in this study indicated that it could be a useful treatment option for Japanese patients with NET.

## Electronic supplementary material

ESM 1 (DOCX 20 kb)
