# Supplementary material for: Phase II study of lanreotide autogel in Japanese patients with unresectable or metastatic well-differentiated neuroendocrine tumors
Source: Invest New Drugs. 2017 May 3;35(4):499–508. doi: 10.1007/s10637-017-0466-8 (PMC5502055; doi:10.1007/s10637-017-0466-8)
Supplement: Supplementary file 1 — (DOCX 20 kb) [file 10637_2017_466_MOESM1_ESM.docx]

**Electronic supplementary material**

**Title**: Phase II study of lanreotide autogel in Japanese patients with unresectable or metastatic, well-differentiated neuroendocrine tumors

**Journal**: Investigational New Drugs

**Authors**: Tetsuhide Ito, Yoshitaka Honma, Susumu Hijioka, Atsushi Kudo, Akira Fukutomi, Akira Nozaki, Yasutoshi Kimura, Fuyuhiko Motoi, Hiroyuki Isayama, Izumi Komoto, Seiichi Hisamatsu, Akihiro Nakajima, Akira Shimatsu

**Corresponding author**: Tetsuhide Ito,Department of Medicine and Bioregulatory Science, Graduate School of Medical Science, Kyushu University,

E-mail: itopapa@intmed3.med.kyushu-u.ac.jp

**eMethods**

**eResults**

**eMethods**

Exclusion criteria:

Patients with Grade 3 neuroendocrine tumors (NETs) were excluded. Patients who received either octreotide acetate, mammalian target of rapamycin inhibitor (mTOR inhibitor), multi-target tyrosine kinase inhibitor (MTK inhibitor), octreotide acetate sustained release formulation, lanreotide acetate sustained release formulation, other study drugs or unapproved drugs, radiotherapy, chemotherapy, radiofrequency ablation or cryoablation, NET-related surgery, or interferon treatment, transcatheter arterial embolization (TAE), or transcatheter arterial chemo-embolization (TACE) were subjected to a 1–24-week washout period. In addition, patients were excluded if they received peptide receptor radionuclide therapy (PRRT), or met the following criteria: baseline (defined as the first day of treatment before treatment administration) aspartate aminotransferase (AST) or alanine aminotransferase (ALT) level ≥2.5-fold the upper limit of normal (ULN) or ≥5-fold the ULN if abnormal hepatic function was attributable to NET, baseline serum creatinine level of ≥2 mg/dL, grade II or higher atrioventricular block, arrhythmia necessitating treatment, other severe cardiac disease, previous or current malignant tumors other than NET or hypersensitivity to somatostatin analogues, and current symptomatic cholelithiasis.

Bioassay:

Serum chromogranin A concentrations were determined centrally every 12 weeks for the 48-weeks period following the start of administration, and every 24 weeks thereafter (SRL Medisearch Inc.,Tokyo,Japan, Kit Cisbio).

Serum anti-lanreotide antibody levels were determined every 4 weeks for the 24-weeks period following the start of administration, and every 12 weeks thereafter for the 48-weeks. Serum anti-lanreotide antibody levels were centrally determined using a validated radioimmunoprecipitation assay (RIPA; SGS Cephac, Saint-Benoît, France). Anti-lanreotide antibody-positivity was evaluated in two stages: screening test and confirmation test. Antibody titers were determined for samples found to be positive in the confirmation test.

Regarding pharmacokinetics evaluations, serum lanreotide concentrations were determined on the day of first administration (before and 2 and 4 h after administration of the study drug) and at 4, 8, 12, 16, and 20 (before and 2 and 4 h after administration of the study drug), 24, 36, and 48 weeks after the start of administration (or at the time of discontinuation). Serum lanreotide concentrations were centrally determined using a validated radioimmunoassay (RIA; Kymos Pharma Services S.L., Barcelona, Spain).

**eResults**

Grade 3 or higher adverse events (AEs)

eTable1. Tabulation of grade 3 or higher adverse events (AEs) (Safety analysis set, N=32)

| SOC ^a） b）^  PT | N = 32 | | |
| --- | --- | --- | --- |
|  | Grade 3 ^c)^ | Grade 4 ^c)^ | Grade 5 ^c)^ |
|  | Number of patients (%) | Number of patients (%) | Number of patients (%) |
| Adverse events | 11 (34.4) | 2 (6.3) | 0 |
| Gastrointestinal disorders | 3 (9.4) | 0 | 0 |
| Abdominal pain | 1 (3.1) | 0 | 0 |
| Diarrhoea | 1 (3.1) | 0 | 0 |
| Abdominal pain upper | 1 (3.1) | 0 | 0 |
| Ascites | 1 (3.1) | 0 | 0 |
| Pancreatitis | 1 (3.1) | 0 | 0 |
| Duodenal stenosis | 1 (3.1) | 0 | 0 |
| Infections and infestations | 1 (3.1) | 0 | 0 |
| Liver abscess | 1 (3.1) | 0 | 0 |
| Metabolism and nutrition disorders | 4 (12.5) | 1 (3.1) | 0 |
| Diabetes mellitus | 1 (3.1) | 0 | 0 |
| Decreased appetite | 1 (3.1) | 1 ( 3.1) | 0 |
| Diabetes mellitus inadequate control | 1 (3.1) | 0 | 0 |
| Hyperglycaemia | 1 (3.1) | 0 | 0 |
| Hypoalbuminaemia | 1 (3.1) | 0 | 0 |
| Investigations | 4 (12.5) | 1 (3.1) | 0 |
| Aspartate aminotransferase increased | 1 (3.1) | 0 | 0 |
| Gamma-glutamyltransferase increased | 3 (9.4) | 0 | 0 |
| Blood glucose increased | 2 (6.3) | 0 | 0 |
| Amylase increased | 1 (3.1) | 0 | 0 |
| Blood bilirubin increased | 0 | 1 (3.1) | 0 |
| Blood sodium decreased | 1 (3.1) | 0 | 0 |
| Haemoglobin decreased | 1 (3.1) | 0 | 0 |
| Lymphocyte count decreased | 1 (3.1) | 0 | 0 |
| Injury, poisoning and procedural complications | 1 (3.1) | 0 | 0 |
| Road traffic accident | 1 (3.1) | 0 | 0 |
| Nervous system disorders | 1 (3.1) | 0 | 0 |
| Hepatic encephalopathy | 1 (3.1) | 0 | 0 |
| Vascular disorders | 3 (9.4) | 0 | 0 |
| Hypertension | 3 (9.4) | 0 | 0 |
| Surgical and medical procedures | 1 (3.1) | 0 | 0 |
| Parathyroidectomy | 1 (3.1) | 0 | 0 |

N=Number of subjects

1. MedDRA version 16.0
2. When a patient had more than one AE, the most severe AE was used for tabulation
3. Adverse events were evaluated using the National Cancer Institute Common Terminology Criteria for Adverse Events (NCI-CTCAE), ver. 4.0.

Antibodies

In this study, 2 patients (6.3%) were positive for anti-lanreotide antibodies. No patients experienced an onset of lack of efficacy and adverse events related to study drug reactions (ADRs) that were presumably attributable to these antibodies, during the study period.

eTable 2. Anti-lanreotide antibody testing and antibody titers

|  | Time point | | Anti-lanreotide antibody test | | Antibody titer |
| --- | --- | --- | --- | --- | --- |
| Patient A | | Baseline | | Negative | - |
|  | | Week 4 | | Negative | - |
|  | | Week 8 | | Negative | - |
|  | | Week 12 | | Negative | - |
|  | | Week 16 | | Positive | 1/1 |
|  | | Week 20 | | Negative | - |
|  | | Week 24 | | Negative | - |
|  | | Week 36 | | Positive | 1/2 |
| Patient B | | Baseline | | Negative | - |
|  | | Week 4 | | Negative | - |
|  | | Week 8 | | Positive | 1/4 |
|  | | Week 12 | | Positive | 1/32 |
|  | | Week 16 | | Positive | 1/32 |
|  | | Week 20 | | Positive | 1/64 |
|  | | Week 24 | | Positive | 1/64 |
|  | | Week 36 | | Positive | 1/256 |
|  | | Week 48 | | Positive | 1/512 |
